# Hypocomplementemic Urticarial Vasculitis Syndrome or Systemic Lupus Erythematosus in Evolution?

**DOI:** 10.7759/cureus.23429

**Published:** 2022-03-23

**Authors:** Vartika Kesarwani, Deep Phachu, Ruchir Trivedi

**Affiliations:** 1 Internal Medicine, University of Connecticut, Farmington, USA; 2 Department of Nephrology, University of Connecticut, Farmington, USA

**Keywords:** chronic urticaria, urticarial vasculitis, mcduffie syndrome, huvs, hypocomplementemia urticarial vasculitis syndrome

## Abstract

Hypocomplementemia urticarial vasculitis syndrome (HUVS) is a rare form of systemic vasculitis which is characterized by the presence of urticaria and hypocomplementemia. The presence of recurrent and chronic urticarial rash is the dominant clinical finding in HUVS. Other manifestations including angioedema, arthritis, gastrointestinal symptoms, ocular inflammation, pulmonary involvement, renal involvement, and central nervous system involvement are also seen. Although the pathophysiology of HUVS is yet to be fully understood, it has been demonstrated that immune complex-mediated injury is the predominant mechanism responsible for severe systemic manifestations; a mechanism of injury similar to systemic lupus erythematosus (SLE). HUVS shared many clinicopathological features with SLE and it is prudent to question whether HUVS is a separate disease entity or SLE in evolution. Herein we present a case of a male patient who was diagnosed with SLE a year after being diagnosed with HUVS.

## Introduction

Hypocomplementemia urticarial vasculitis syndrome (HUVS) is a rare form of systemic vasculitis characterized by the presence of urticaria and low serum complement levels. It typically presents during the fourth to fifth decade of life with an 8:1 female predominance [1]. HUVS affects multiple organ systems and can present as leukocytoclastic vasculitis, angioedema, laryngeal edema, pulmonary involvement, arthralgias and arthritis, glomerulonephritis, and uveitis [1]. Pulmonary involvement is seen in approximately 20-50% of the cases and is the leading cause of mortality and morbidity in HUVS [2]. The majority of the patients (90-100%) have elevated levels of anti-C1q antibodies [3]. HUVS can occur either alone or in association with other autoimmune disorders, such as systemic lupus erythematosus (SLE) [4]. HUVS shares many clinical and pathological features with SLE. It has been demonstrated that HUVS is present in 7-8% of SLE patients and 54% of HUVS patients are diagnosed with SLE in the follow-up period [2]. Therefore, several authors have emphasized that HUVS is likely a precursor to SLE. Herein, we present a case of HUVS in a male patient with a history of urticaria and angioedema who was later diagnosed with SLE during the follow-up period.

## Case presentation

**Figure 1:**
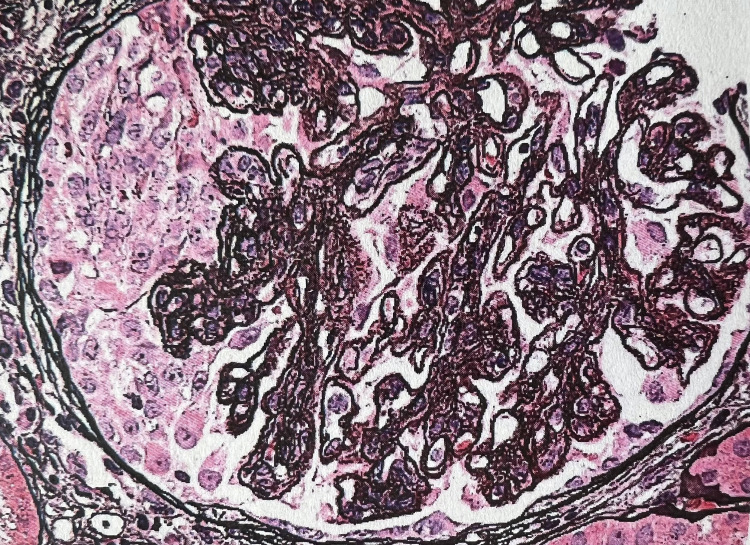
Membranoproliferative glomerulonephritis and glomerular crescent formation.

**Figure 2:**
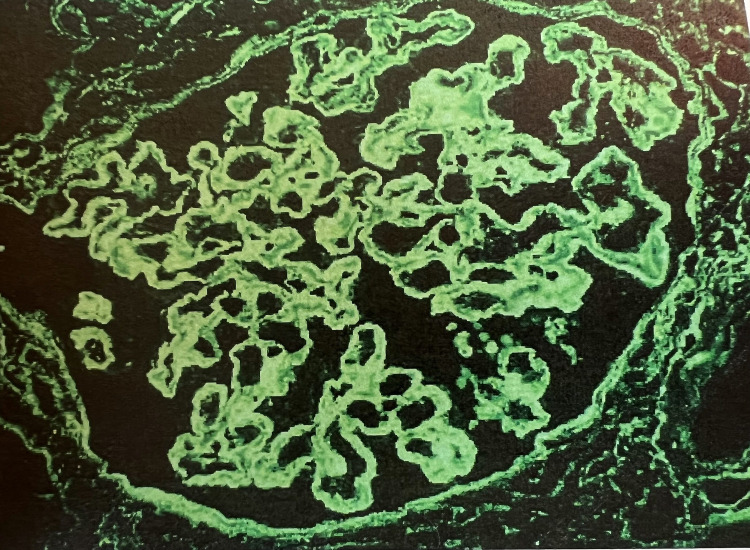
Full house staining pattern with IgG deposits on immunofluorescence.

Our patient is a 60-year-old Jamaican male with chronic urticaria and angioedema of five years duration. He did not have a history of angiotensin-converting enzyme (ACE) inhibitor use. He had been following up with an allergist-immunologist and was treated with an antihistamine, but with poor response. During the year prior to referral to our clinic, he required several hospital admissions for shortness of breath and bilateral lower extremity swelling and he was found to have nephrotic syndrome. His workup at that time was significant for serum creatinine of 1.2 mg/dL, 7.5 g of proteinuria, and serum albumin of 2.0 g/dL. Serological workup demonstrated low complement levels-C4 12 mg/dL (15-45 mg/dL), and C3 42 mg/dL (83-177 mg/dL) along with an elevated anti-C1q antibody titer of 158 (>40 positive). Other serological workups including antinuclear antibody (ANA), anti-double-stranded DNA (anti-dsDNA), anti-smith (anti-Sm), lupus anticoagulant, anti-ribonucleoprotein (anti-RNP) antibodies, cryoglobulin level, hepatitis B and C panel were negative. He subsequently underwent a percutaneous kidney biopsy which demonstrated the presence of diffuse endocapillary and focal extra capillary proliferative and membranous glomerulonephritis (Figure 1) with “full house staining” pattern on immunofluorescence (Figure 2), suggestive of lupus nephritis class IV and V versus HUVS. He met both major and 3 minor Schwartz criteria and a diagnosis of hypocomplementemic urticarial vasculitis was established. He was started on furosemide for lower extremity swelling, losartan for proteinuria, and prednisone 40 mg daily for glomerulonephritis. However, he continued to have anasarca, with worsening renal function, nephrotic range proteinuria of 7.1 g/day, and persistently depressed complement levels-C3 of 29 mg/dL and C4 of 11 mg/dL. Mycophenolate mofetil (MMF) 1.5 g twice daily was subsequently added, with stabilization of serum creatinine and gradual improvement in proteinuria and anti-C1q antibody titers.

Over the following few months, his shortness of breath gradually worsened. His pulmonary function test which was previously unremarkable demonstrated a precipitous decline in forced expiratory volume (FEV1)/forced vital capacity (FVC) to a ratio of 26.55% with no bronchodilator reversibility, findings consistent with severe chronic obstructive pulmonary disease (COPD). A computed tomographic (CT) scan of the chest revealed the presence of diffuse pan acinar emphysema. Given that he is a lifetime non-smoker, and his alpha-1 antitrypsin levels were normal, his obstructive lung disease was likely associated with his known diagnosis of HUVS. He also developed other systemic manifestations of HUVS including fatigue, arthralgia, and arthritis during the same time.

**Figure 3:**
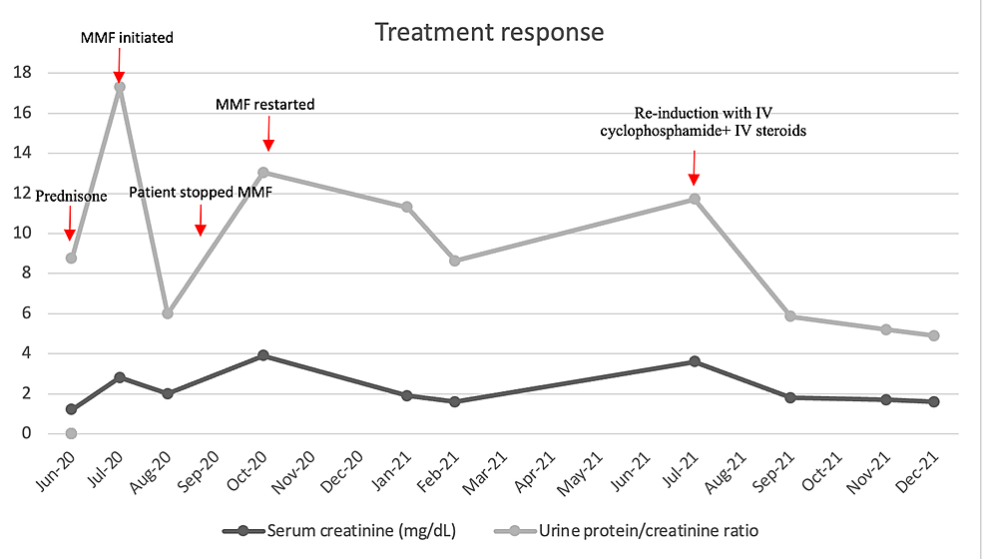
Timeline and treatment response to different pharmacological agents. MMF: mycophenolate mofetil

On follow-up after 10 months of MMF at SLE induction dosing, he demonstrated only partial complement recovery as he had ongoing hematuria and persistent nephrotic range proteinuria of approximately 4 g/day. His ANA which was previously negative resulted in positive (titer 1:80) on repeat workup, which along with “full house” staining pattern on kidney biopsy raised the possibility of a lupus-like process in evolution. MMF was discontinued and reinduction therapy with intravenous (IV) cyclophosphamide and corticosteroids at Euro-lupus dosing (cyclophosphamide 500 mg every 14 days for six doses and IV corticosteroids 250 mg for three doses followed by prednisone 10 mg daily) was given. Following the completion of cyclophosphamide induction therapy, his serum creatinine stabilized, complement levels normalized for the first time since his diagnosis, and his hematuria and proteinuria also significantly decreased (Figure 3). He has since been started on maintenance therapy with rituximab and oral hydroxychloroquine.

## Discussion

HUVS or McDuffie syndrome is an uncommon form of autoimmune small-vessel vasculitis that is characterized by urticaria and persistent hypocomplementemia. It is a rare disease with a reported incidence of 0.5/100,000 persons [5]. The disease affects females more than males (female: male ratio of 8: 1) and typically presents during the fourth to fifth decade of life [1].

The presence of recurrent and chronic urticarial rash is the dominant clinical finding in HUVS that frequently precedes the development of other systemic manifestations. Angioedema, arthritis, gastrointestinal symptoms including abdominal pain, nausea, vomiting, and diarrhea [6,7] and ocular inflammation are other frequently observed findings [1]. Pulmonary involvement is seen in the form of moderate to severe COPD and asthma and it is seen in approximately 20-50% of the cases [1,8-10]. Central nervous system involvement, although uncommon, may occur in the form of pseudotumor cerebri, cranial nerve palsies, and aseptic meningitis.

Renal involvement in HUVS is seen in up to 50% of the patients. Hematuria and sub-nephrotic range proteinuria are the most common presenting features. Mild nonprogressive renal disease is typical of HUVS, but some patients may have a rapid progression to end-stage renal disease. The mechanism of injury and the pattern of renal involvement on kidney biopsy is indistinguishable from that of lupus nephropathy. Mesangioproliferative and membranoproliferative glomerulonephritis are the most common histological pattern, followed by membranous nephropathy [11].

HUVS shares many clinicopathological features with SLE. It has been demonstrated that the clinical manifestations of HUVS are related to immune complex-mediated injury, a mechanism similar to SLE. Elevated levels of anti-C1q antibody, a finding seen in the majority of patients in HUVS (90-100%) binds to the collagen domain of the C1q resulting in the formation of immune complexes that deposit on the vascular lumina. This immune complex deposition triggers the activation of the complement pathway which results in damage of the endothelium and surrounding tissues [12].

The diagnosis of HVUS is based on the fulfillment of the Schwarz criteria (Table 1) [13] which includes clinical and laboratory findings similar to the American College of Rheumatology (ACR) diagnostic criteria for SLE [13]. However, the ACR criteria for the diagnosis of SLE mandates the presence of ANA with a titer of >= 1:80 to establish the diagnosis of SLE while the presence of these antibodies rules out the diagnosis of HUVS [11]. Therefore, patients who would otherwise fulfill the criteria for SLE are diagnosed as HUVS in the absence of ANA positivity. Our patient was initially found to have negative ANA and was diagnosed with HUVS. However, during the one-year follow-up, his ANA resulted positive with a titer of 1:80 which in association with the “full house” effect seen on the kidney biopsy and low complement levels established the diagnosis of SLE. The findings of our case are similar to other cases described in the literature where patients with clinical features consistent with SLE were initially diagnosed as HUVS and subsequently found to have antibody positivity leading to a diagnosis of SLE [4,5]. HUVS was present in about 7-8% patients with SLE and around 54% of the patients with HUVS were subsequently diagnosed with SLE in the follow-up period [5]. Considering the abovementioned evidence, it is prudent to question whether HUVS is a separate clinical entity or SLE in evolution.

**Table 1:** Schwartz criteria for diagnosis of hypocomplementemia urticarial vasculitis syndrome (HUVS).

Schwartz Criteria	
Major criteria	Chronic urticaria for more than 6 months
	Hypocomplementemia
Minor criteria	Arthralgias or arthritis
	Ocular inflammation
	Glomerulonephritis
	Abdominal pain
	Low C1q with positive anti-C1q antibodies

There is no clear consensus regarding the approach to the management of HUVS. In patients with severe systemic disease, high-dose glucocorticoids are the treatment of choice. However, most patients develop relapses, and a combination of steroids with other immunosuppressant/immunomodulatory agents including cyclophosphamide, azathioprine, MMF, and cyclosporine are often used. In our case, the patient responded well to treatment with corticosteroids and cyclophosphamide at Euro-lupus dosing used for lupus nephritis. Through our case, we also highlight that since the mechanism of renal injury in HUVS is indistinguishable from SLE, the treatment approach should be guided by the histopathological features seen on renal biopsy. This approach may help delay the progression of the disease and avoid catastrophic consequences.

## Conclusions

HVUS represents a rare clinical entity in a patient population with frequent hospitalizations. Its clinical presentation and management remain yet to be fully elucidated. Our case highlights the importance of recognition of HUVS and its association with SLE. Clinicians should maintain a high degree of suspicion in order to ensure timely implementation of the appropriate treatment and to avoid catastrophic consequences.
